# Activation of the C3a–C3aReceptor-axis is associated with endothelial dysfunction and glycocalyx damage in ST-elevation myocardial infarction

**DOI:** 10.1007/s00395-026-01181-w

**Published:** 2026-04-28

**Authors:** Carl Vahldieck, Samuel Löning, Constantin Hamacher, Benedikt Fels, Tanja Svensson, Bettina Rudzewski, Joachim Weil, Kristina Kusche

**Affiliations:** 1 Department of Anesthesiology and Intensive Care Medicine, University Medical Centre Schleswig-Holstein Campus Luebeck, Luebeck, Germany; 2 Institute of Physiology, University of Luebeck, Luebeck, Germany; 3 DZHK (German Research Centre for Cardiovascular Research), Partner Site North, Luebeck, Germany; 4 Department of Cardiology, Medical Clinic II, Sana Kliniken Luebeck, Luebeck, Germany

**Keywords:** ST-elevation myocardial infarction (STEMI), Complement system, C3a:C3aReceptor signaling, Endothelial glycocalyx, Rac1 activation, Endothelial dysfunction

## Abstract

Complement activation is an early event in ischemia–reperfusion injury during ST-elevation myocardial infarction (STEMI) and drives endothelial dysfunction via glycocalyx (eGC) degradation. While downstream fragments such as C5a contribute to vascular injury, the role of the early anaphylatoxin C3a remains unclear. This study delineates the effects of the C3a:C3a-Receptor-axis on endothelial function, cytoskeletal dynamics, and eGC integrity. Sixty-four first-time STEMI patients and sixty-four age- and sex-matched healthy controls were enrolled. Patients were stratified into quartiles based on serum C3a concentrations, and comparisons were performed between the lowest vs. highest quartiles as well as between all STEMI patients vs. controls. Inflammatory and glycocalyx parameters were assessed via ELISA, AFM nanoindentation, and monocyte adhesion assays. NO bioavailability was measured chemiluminescence-based. C3a-receptor-antagonists (SB290157 and JR14a), C5a-Receptor1-antagonism (PMX53), as well as Rac1-Inhibition (NSC23766) were used to verify pathway specificity and downstream signaling involvement. High C3a levels were associated with marked endothelial injury: eGC height was reduced (− 44%; p < 0.001), cortical stiffness increased (+ 35%; p < 0.001), and shedding of Syndecan-1 and heparan sulfate was elevated (+ 203%, p < 0.001; + 181%, p < 0.01). NO bioavailability decreased by 34% (p < 0.05). C3a correlated inversely with eGC height (r =  − 0.736) and positively with Syndecan-1 (r = 0.856). Treatment with recombinant C3a (250 ng/mL) induced cortical stiffening (+ 10.8%; p < 0.001), eGC loss (− 24.7%; p < 0.001), actin polymerization (+ 27.9%; p < 0.001), Rac1 activation (p < 0.05), reduced NO (− 38%; p < 0.05), and increased monocyte adhesion (+ 37%), all reversed by both C3a-Receptor-inhibitiors and by Rac1-inhibition. C3a:C3a-Receptor signaling drives Rac1-mediated cytoskeletal stiffening, eGC degradation, NO reduction, and leukocyte adhesion, promoting endothelial dysfunction in STEMI in both macrovascular and microvascular endothelial cells. This pathway represents a potential therapeutic target to mitigate complement-mediated vascular injury in acute myocardial infarction.

## Introduction

The activation of the complement system plays a central role in the pathophysiology of myocardial damage in acute myocardial infarction (AMI) and ST-elevation myocardial infarction (STEMI). Shortly after an ischemic event, damage-associated molecular patterns and altered surface structures of necrotic cardiomyocytes are released, activating several innate immune pathways, including the classical, lectin, and alternative pathways of the complement system [5, 22, 53]. In particular, the complement protein C3 is cleaved, further leading to pro-inflammatory fragments such as C3a and C3b, which recruit immune cells and intensify a local inflammatory response [5, 49].

Complement activation in AMI mediates inflammatory damage to the myocardium and vasculature. It is associated with larger infarct size and poor clinical outcomes [7, 23, 33]. Activation of the complement cascade also initiates proinflammatory signaling pathways in endothelial cells, leading to increased adhesion molecules and neutrophil granulocyte recruitment [15, 55]. The resulting excess of C3a/C3b contributes to complement-mediated opsonization, cell lysis, and amplification of inflammation [12, 25, 30]. AMI-induced tissue damage from ischemia–reperfusion causes immediate activation of the complement system, which begins locally at the endothelium and disrupts vascular homeostasis [18, 40]. Earlier steps in the cascade, particularly the formation and hyperfunction of C3 convertase, lead to the cleavage of C3 into C3a and C3b and the deposition of C3 fragments on the endothelial surface, marking the vessel for further pro-inflammatory reactions [24, 40].

Beyond cardiomyocyte necrosis, myocardial infarction is increasingly recognized as a disease of the coronary microcirculation. Contemporary concepts emphasize that microvascular obstruction, endothelial swelling, capillary rarefaction, and intramyocardial hemorrhage critically determine infarct expansion, impaired reperfusion, and long-term ventricular remodeling. These microvascular alterations are not merely secondary phenomena but actively drive ischemia–reperfusion injury, adverse remodeling, and progression to ischemic heart failure, even in the presence of successful epicardial reperfusion. Importantly, inflammatory activation of the endothelium has been identified as a key upstream event linking myocardial injury to microvascular dysfunction [27, 36].

Although cardiomyocyte damage is the primary concern during an acute myocardial infarction (AMI), significant damage also occurs to the vascular endothelium, particularly to the endothelial glycocalyx (eGC) [7, 10, 25]: the eGC is a negatively charged, mesh-like, hydrated structure that covers the luminal surface of endothelial cells. Proteoglycans (syndecans and glypicans), which have glycosaminoglycans (heparan sulfate and hyaluronic acid) bound to them, are the main contributors to the glycocalyx's structure and function. The eGC, together with the endothelial cellular cortex (an actin-rich layer located 50 to 150 nm beneath the plasma membrane), forms a responsive functional unit. The eGC and endothelial cortex are highly dynamic structures that can adapt their mechanical properties (e.g., stiffness and/or height) in response to environmental changes in the bloodstream. This responsiveness is imperative for proper endothelial functioning [38, 44, 45]. Alterations in the mechanical properties of the eGC or the endothelial cellular cortex can lead to impaired secretion of vasoactive substances, such as nitric oxide (NO), which is considered a cornerstone in the development of endothelial dysfunction. Thus, the eGC and its underlying cellular cortex act as the epicenter of the pathophysiology of various cardiovascular diseases [20, 21, 29, 37, 50]. Recent comprehensive reviews have highlighted the endothelial surface layer, including the glycocalyx, as a critical regulator of coronary microvascular homeostasis. The glycocalyx integrates mechanotransduction, barrier integrity, and anti-inflammatory signaling and thereby governs capillary perfusion, shear stress sensing, and nitric oxide bioavailability. Acute glycocalyx degradation during myocardial infarction has been associated with increased microvascular permeability, leukocyte plugging, impaired capillary recruitment, and no-reflow phenomena. Thus, disruption of the endothelial glycocalyx represents a central mechanistic link between inflammation, endothelial dysfunction, and coronary microvascular injury [28, 32].

Although myocardial injury and activation of the vasculature have already been demonstrated in the course of AMI. Up to now there is only few information regarding the impact of the complement anaphylatoxin C3a-mediated endothelial injury and function and eGC integrity during the event.

Recently, our group published data concerning the role of the C5a/C5a-Receptor-1-axis on eGC damage with subsequent endothelial dysfunction [46]. However, inhibition of the C5a/C5a-Receptor-1-axis could not completely prevent AMI-induced eGC damage leading to endothelial dysfunction and exaggerated leucocyte adhesion, indicating that the exclusive use of anti-C5 and other downstream factors of the common complement pathway cannot explain the full influence of complement-related changes to the endothelium [23, 33, 47]. In addition to C3a, the complement anaphylatoxin C5a has been identified as a potent pro-inflammatory effector with significant impact on vascular integrity in the context of myocardial infarction. C5a levels are elevated in STEMI patients and have been shown to correlate with endothelial glycocalyx degradation, increased cortical stiffness, reduced nitric oxide bioavailability, and enhanced monocyte adhesion, pointing to a substantial contribution to endothelial dysfunction independent of C3a signaling. Mechanistically, C5a engages the C5a receptor 1 (C5aR1/CD88) on endothelial and immune cells, leading to RhoA-mediated cytoskeletal remodeling and upregulation of adhesion molecules, thereby promoting vascular inflammation and impaired microvascular function. These findings suggest that parallel activation of C5a may act synergistically with C3a to exacerbate vascular injury in the setting of acute myocardial infarction [46].

Thus, it is likely that activation of factors upstream to C5 is associated with early complement signaling pathways that may contribute to acute cell death and chronic inflammation in the context of post-AMI endothelial dysfunction. Importantly, recent reviews have repositioned the complement system as a central driver of coronary microvascular injury rather than a purely myocardial effector mechanism. Early complement activation products, particularly upstream components such as C3a, have been implicated in endothelial barrier breakdown, leukocyte–endothelial interactions, microvascular inflammation, and impaired vasomotor regulation. These processes critically contribute to microvascular obstruction and persistent ischemia after reperfusion. Notably, upstream complement signaling has been proposed to act independently of terminal complement activation, potentially explaining the limited efficacy of downstream complement inhibition strategies observed in experimental and clinical settings [2, 36].

Although there is accumulating evidence that the anaphylatoxin C3a contributes to endothelial injury and that complement-driven damage to the endothelial glycocalyx occurs in the context of AMI [46, 52], the precise molecular mechanisms linking early C3 activation to glycocalyx degradation and endothelial dysfunction remain incompletely understood [24, 35]. Therefore, targeted blockade of the C3a receptor and focused mechanistic studies are needed to clarify causality and evaluate a possible therapeutic potential of C3a antagonism.

The aim of this study was to evaluate the effects of the complement anaphylatoxin C3a on the nanomechanical properties of the eGC and cellular cortex in the context of AMI. In the present study we report the changes of endothelial function and eGC condition after AMI-induced C3a elevation. Using the AFM nanoindentation technique we evaluate the contribution of the C3a/C3a-receptor-axis on eGC integrity and endothelial function as well as on leucocyte–endothelium-interaction, and investigate the effects of C3aR-antagonist (SB290157) administration on eGC damage, endothelial inflammation and endothelial function.

## Methods

### Study population

Sixty-four patients with first-time ST-elevation myocardial infarction (STEMI) were recruited at the University of Lübeck in collaboration with the Department of Cardiology and Angiology at Sana-Kliniken-Lübeck Hospital (Germany) in accordance with the Declaration of Helsinki and with the approval of the local ethics committee (case: 19–310). Patients with STEMI who received percutaneous coronary intervention (PCI) as initial treatment within the first 120 min after diagnosis were randomly enrolled in the study after obtaining their written consent. Serum samples were collected during emergency PCI (STEMI cohort). Sixty-four volunteers of the same age and sex without cardiovascular comorbidities served as the control group (CTR cohort). Serum samples from patients and control subjects were immediately processed according to Brandwijk et al. [8]. Patients who underwent cardiopulmonary resuscitation were excluded, as were patients who died during or after PCI. Other exclusion criteria included age under 18, pregnancy, or lack of consent.

### Atomic force microscopy

#### Nanoindentation

The height and stiffness of the eGC and the cortical stiffness of HUVEC were determined using the AFM-based nanoindentation technique as described previously [44]. The indentation measurements were performed on living confluent cells at 37 °C in a HEPES-buffered solution. (Standard composition in mmol/L: 140 NaCl, 5 KCl, 1 MgCl2, 1 CaCl2, 5 glucose, and 10 HEPES, pH 7.4). A Nanoscope Multimode8 AFM (Veeco, Mannheim, Germany) was used to determine cortical stiffness. A Nanowizard4 (JPK BioAFM Business, Berlin, Germany) was used to measure the nanomechanical properties of HUVEC eGCs and for measurements on ex vivo mouse aortas.

In short, a laser beam was directed onto the back of a gold-coated triangular cantilever (Novascan Technologies, Boone, NC, USA) with an attached spherical tip (diameter 10 μm) and a nominal spring constant of 10 pN/nm (for eGC) and 30 pN/nm (for cortical stiffness). The cantilever indents the surface of the endothelial cells with a force of 0.5 nN and 3 nN, respectively. The reflection of a laser beam is used to quantify the deflection of the cantilever. The height of the eGC can be calculated if the cantilever force, piezo displacement, and deflection sensitivity are known. A total of 150 to 300 individual cells were measured for each experimental condition. The data was acquired using Research NanoScope version 9.20 (64-bit; Bruker Nano GmbH) and calculated using Protein Unfolding and Nano-Indentation Analysis Software (PUNIAS 3D; Version 1.0; Release 2.3; Copyright 2009).

#### Single-cell force spectroscopy and quantitative monocyte adhesion measurements

The adhesion forces between the endothelial surface and monocytes were quantified using single-cell force spectroscopy and monocyte wash-away assays, as described elsewhere [46]. Human monocytes were isolated from the blood of healthy donors using the S-pluriBead Maxi Reagent Kit (pluriSelect Life Science, Leipzig, Germany; catalog number 70-50010-12) according to the manufacturer's instructions.

For single-cell force spectroscopy, a single human monocyte was bound to the AFM cantilever to measure the adhesion forces between the monocyte and HUVEC monolayers. Measurements were performed using the Nanowizard4 CellHesion module (JPK BioAFM Business, Berlin, Germany). Arrow TL-2 tipless cantilevers (NanoAndMore GmbH, Wetzlar, Germany) were incubated in Corning Cell-Tak (Fisher Scientific GmbH, Schwerte, Germany; catalog: 10317081) for 20 min prior to all experiments in order to bind the monocytes to the cantilever. To quantify the adhesion forces between the monocytes and the endothelial cells, the monocytes were attached to a cantilever and brought into contact with the endothelial surface for 1 s at a constant setpoint of 0.5 V and then pulled away to obtain force-distance/adhesion curves. Both, the maximum adhesion force (in µN) between monocytes and the endothelial surface and the adhesion energy (in µJ) were measured and analyzed using JPK data processing software version 7.0.112 (Bruker Nano GmbH, Berlin, Germany). In addition, the interaction between monocytes and endothelial cells was quantified using monocyte wash-away assays. For this purpose, monocytes were labeled with an Alexa Flour 488 anti-human CD14 antibody (25:1000, Biolegend, San Diego, CA, USA; catalog: 367130) and added to a confluent HUVEC monolayer for 4 h. To remove non-adherent monocytes, the cells were carefully washed four times with PBS according to a standardized protocol. HUVEC and adherent monocytes were fixed with 4% paraformaldehyde and subjected to fluorescence microscopy for further analysis.

### Enzyme-linked immunosorbent assay

The concentrations of complement anaphylatoxins C3a, C5a, and the soluble glycocalyx components syndecan-1, heparan sulfate, and hyaluronan were quantified using enzyme-linked immunosorbent assay (ELISA) according to the manufacturer's instructions (C3a and C5a: Thermo Fisher Scientific, Hamburg, Germany; catalog C3a: BMS2089; catalog C5a: BMS2088/Syndecan-1: Human CD138 ELISA kit, Diaclone Research, Besançon, France; catalog: 950.640.192/Heparan Sulfate: Human Heparan sulfate Proteoglycan (HSPG) ELISA Kit, MBS, San Diego, CA, USA; catalog: MBS2023323/Hyaluronan: Hyaluronan Quantikine ELISA Kit, R&D Systems, Minneapolis, MN, USA; catalog: DHYAL0).

### Nitric oxide measurements

The concentrations of NO products [nitrites (NO_2_^−^) and nitrates (NO_3_^−^)] were determined using the Sievers Nitric-Oxide Analyzer 280-i (NOA-280i; GE Water & Process Technologies, Analytic Instruments; Boulder, CO, USA) chemiluminescence detector. The assay is based on the reduction of all nitrates and nitrites into NO by vanadium(III)-chloride (VCl_3_), as described in detail before[45].

In short, NO reacts with ozone (O_3_) to form nitrogen dioxide (NO_2_), which is sensitively detected by its chemiluminescence. All samples were deproteinized by ethanol precipitation prior to analysis. For this purpose, the cell culture supernatants and patient sera were diluted 1:3 with chilled pure ethanol (0 °C). After a precipitation time of 45 min, the samples were centrifuged at 14,000 × g for 15 min and the supernatant was used for the experiment. NO concentrations were analyzed by injecting 50 µl duplicates of each sample. Concentrations were calculated using the manufacturer's NO Analysis Software for Liquid (Version 3.21/Liquid, GE Water & Process Technologies, Analytic Instruments; Boulder, CO, USA).

### Cell isolation and culture

To obtain a clear picture of the effects of stimulation with recombinant complement anaphylatoxins or STEMI serum on the vascular endothelium, different endothelial cell lines were used for the experiments. These included primary endothelial cell lines (HUVEC and HCMEC) as well as immortalized human vascular endothelial cells (EA.hy926 endothelial cells): primary human umbilical vein endothelial cells (HUVEC) were isolated as previously described in detail [44] and cultured in Gibco Medium 199 supplemented with 10% fetal calf serum (FCS; Gibco, Carlsbad, CA, USA), 1% penicillin/streptomycin (100 U/mL, 100 mg/mL; Gibco, Carlsbad, CA, USA), 1% large vessel endothelial supplement (Gibco, Carlsbad, CA, USA), and heparin (5000 U/mL; Biochrom, Schaffhausen, Switzerland) at 37 °C, 21% O_2_, and 5% CO_2_. The umbilical cords were donated by patients who gave birth at Marien-Krankenhaus Lübeck and the University Medical Center Schleswig–Holstein Campus Lübeck (approved by the local ethics committee, cases: 18-325 and 2023-520_1).

Immortalized Human Vascular Endothelial Cells (EA.hy926 Endothelial Cells: kindly provided by Cora-Jean S. Edgell, University of North Carolina, Chapel Hill, NC, USA) were cultured in 12.5 cm^2^ Falcon tissue culture flasks (Corning Inc., Corning, NY, USA; catalog: 353107) until they reached confluence. The cells were cultured in Dulbecco's modified Eagle's medium (DMEM; Thermo Fisher Scientific, Hamburg, Germany; catalog: 41966–029), supplemented with FCS (10%) and penicillin/streptomycin (100 U/mL, 100 mg/mL), at 37 °C, 21% O_2_, and 5% CO_2_.

Primary Human Cardiac Microvascular Endothelial Cells (HCMEC; Sigma Aldrich, Hamburg, Germany; catalog: C-12285) were cultured in PromoCell endothelial cell growth medium including basal medium and supplement mix (Sigma Aldrich, Hamburg, Germany; catalog: C-22020) at 37 °C, 21% O_2_, and 5% CO_2_.

The endothelial cell monolayers were stimulated with the complement anaphylatoxin C3a (Merck, Darmstadt, Germany; catalog: 204881). Various concentrations of C3a (50, 250, 500, and 1000 ng/mL) and various stimulation durations (0, 30, 60, and 120 min, as well as 24 h) were first tested for their effect on cortical stiffness. Further, HUVEC and HCMEC were stimulated with 250 ng/mL C3a with or without the (i) C3a receptor antagonist (C3aRA) SB290157 (Sigma Aldrich, Hamburg, Germany; catalog: 559410) [concentration 1 µg/mL (1:1000)], (ii) C3aRA JR14a (MedChemExpress, NJ, USA; catalog: HY-138161) [concentration 100nM], (iii) C5a Receptor-1 antagonist (C5aRA) PMX53 (Sigma Aldrich, Hamburg, Germany; catalog: 533683) [concentration 1 μg/mL (1:1000)], (iv) Rac1-GTPase-Inhibitor (Rac-I) NSC23766 (Sigma Aldrich, Hamburg, Germany; catalog: SML0952) [concentration: 50 µM] for 24 h. Cells were treated with the Rac-I to assess the functional necessity of Rac1 activity downstream of C3a/C3aR signaling. The inhibitor blocks Rac1 activation by preventing Rac1-GEF interaction, thereby inhibiting Rac1-dependent actin dynamics and allowing evaluation of the contribution of Rac1 to cortical stiffening, glycocalyx integrity, and nitric oxide regulation.

In addition, endothelial cells were stimulated for 24 h with 10% serum from patients (STEMI) or healthy donors (CTR) (instead of FCS) with or without both C3aR-Antagonists, C5aRA and RAC-I.

### Wound healing assay

To investigate the migration characteristics of HUVEC monolayers after treatment with C3a or stimulation with STEMI serum, wound healing assays were performed. For wound closure experiments, EA.hy 926 endothelial cell monolayers were scratched with a sterile 200-μL pipette tip and detached cells were washed away with PBS. Stimulation with C3a or STEMI vs. CTR serum ± C3aR antagonist (SB290157) was performed in a HEPES-buffered solution. After scratching, the culture flasks were placed in a flask warmer set to 37 °C.

Wound closure was analyzed using time-lapse video microscopy with an Olympus CKX53 microscope (EVIDENT Europe GmbH, Hamburg, Germany) with a 10 × objective lens. Images were captured every 5 min for 24 h with a VWR VisiCam 5 (VWR International GmbH, Darmstadt, Germany) using OPTIKA Vision software (version 2.13; OPTIKA S.r.l., Ponteranica, Italy). To quantify cell migration characteristics (wound closure in %), the images were analyzed using an ImageJ plugin (ImageJ software version 1.52a; NIH, Bethesda, MD; https://imagej.net/ij/download.html, last accessed September 28, 2025) for high-throughput image analysis of in vitro scratch wound healing assays developed by Suarez-Arnedo et al. [30]. The wound closure rate was also normalized to the cell growth rate (µm/h) to make all data comparable, as the speed of the cell front is independent of the initial gap width, the method used to create the gap, the gap orientation, the microscopy settings, and the magnification of the objective lens. To do this, the wound closure rate (surface area per time) was determined by creating a graph showing the area covered by cells per time and defining the linear part of the curve (trend line calculation). The slope of the trend line corresponds to the growth rate. Finally, to calculate the cell front velocity, the wound closure velocity was divided by the length of the cell front (in µm). (For more information, see the ibidi website on Applications of Wound Healing and Migration Assays; ibidi GmbH, Gräfelfing, Germany; https://ibidi.com/content/280-principle-wound-healing-and-migration, last accessed on September 28, 2025).

### Fluorescence staining and microscopy

Fluorescence staining and microscopy of cortical F-actin and eGC components were performed as previously described [44]. Briefly, HUVECs were fixed with either 4% paraformaldehyde or 0.1% glutaraldehyde for subsequent staining. Cortical F-actin was stained with phalloidin tetramethylrhodamine (10 mg/mL; Sigma Aldrich) after the cells had been permeabilized for 10 min with 0.1% Triton X-100 (Sigma Aldrich; catalog: T8787-50ML). The coverslips were covered overnight at 4  °C with Dako mounting medium (Dako, Carpinteria, CA; catalog: GM30411-2). Wheat germ agglutinin (WGA; Alexa-Fluor488 conjugate; Thermo Fisher, Waltham, MA) was used as an overview stain for eGC components. After fixation, the cells were incubated with 1:500 dilutions of WGA and mounted overnight. For immunostaining of syndecan-1 (CD138), the fixed cells were incubated with the primary antibody (1:100, mouse anti-human CD138; monoclonal antibody; Bio-Rad, Hercules, CA; catalog: MCA2459). After incubation, the coverslips were incubated with the secondary antibody (1:400, goat anti-mouse conjugate Alexa-488; Invitrogen, Carlsbad, CA; catalog: A28175) and covered with Dako mounting medium containing Hoechst solution (1.5 mg/mL; Sigma Aldrich, Hamburg, Germany; catalog: 94403) to stain the cell nuclei.

Images were rendered with a Keyence fluorescence microscope BZ9000 (Keyence Corp., Osaka, Japan; magnification 60 ×) using the BZ Viewer/Analyzer-II (software version 2.2; Keyence Corp.). Images and stacks of WGA and phalloidin staining were analyzed for fluorescence intensity (in arbitrary units) using ImageJ software. For analyzing the amount of syndecan-1 per cell relative to control, fluorescence-dot nuclei colocalization was measured using YT Evaluation software (Version 2.1.12014; 64 bit; Synentec, Elmshorn, Germany).

### Small G-protein activation assays

The intracellular concentrations of GTP-bound Rac1 and RhoA GTPases were measured by using colorimetric G LISA activity measurements (G-protein ELISA assays; Cytoskeleton, Denver, CO, USA.; catalog: Rac1: BK128; RhoA: BK124). HUVEC were serum starved for 24 h and treated either with C3a or STEMI vs. CTR serum ± C3aR antagonist (SB290157). After processing, cell lysates were subjected to the G-LISA according to the manufacturer’s instructions. The final reaction absorbance was measured using a Mitras LB940 microplate reader (Berthold Technologies, Bad Wildbad, Germany). Absorbance was detected at 490 nm. Presented data are background subtracted.

### Statistical analysis

The data were analyzed using IBM SPSS Statistics for Windows (IBM Corp., released 2025, version 31.0.0.0 Armonk, New York, NY, USA) and the 2D graphics and biostatistics software GraphPad PRISM (version 8.4.2, GraphPad Software Inc., San Diego, CA, USA). GraphPad PRISM was also used to create the figures.

Gaussian distribution was determined using a D'Agostino–Pearson omnibus normality test. Data were checked for outliers using the ROUT outlier test based on the false discovery rate (FDR; Q value = 1%) prior to applying statistical tests. Outliers were excluded from further analysis.

Differences between the two groups were analyzed using the student’s t-test (parametric values) or the Mann–Whitney U test (nonparametric values). Group differences at the nominal scale level were measured using Cramer's V. Categorical variables were compared using the Chi-square test. Differences between three or more groups were analyzed using a one-way analysis of variance (ANOVA) for parametric values or the Kruskal–Wallis test for nonparametric values, followed by a post hoc analysis using Tukey's multiple comparisons test. Correlations at the ordinal scale level were measured using Spearman and at the metric scale level using Pearson correlations (rho (r)).

With a sample size of n = 64, a statistical power of 0.8, and a significance level of α = 0.05, a correlation of r = 0.344 was required for a significant result.

The STEMI patients were retrospectively divided into cohorts: the concentration of C3a in the patient serum was determined using ELISA. The patient group with a concentration of C3a in the lowest quartile (n = 16) is referred to below as LOW, and the group of patients with a concentration in the highest quartile (n = 16) as HIGH.

Differences were considered statistically significant if p < 0.05 (*: p < 0.05, **: p < 0.01, ***: p < 0.001). The data are presented as mean ± standard deviation (m ± SD).

## Results

### Baseline demographic and disease characteristics of patient cohorts

This study randomly enrolled 64 patients with first-time ST-elevation myocardial infarction (STEMI) treated by primary PCI. Based on serum C3a concentrations, patients were stratified into quartiles, with the lowest and highest quartiles defining the LOW (n = 16) and HIGH (n = 16) C3a cohorts, respectively. Serum from healthy donors served as controls (CTR; mean C3a concentration = 19.0 ± 7.6 ng/mL). The overall STEMI population was predominantly male (76%) with a high prevalence of cardiovascular risk factors, including hypertension (79%), hyperlipidemia (89%), and active smoking (62%). While traditional risk factors were comparable between LOW and HIGH C3a cohorts, markers of inflammation, endothelial injury, and complement activation differed substantially.

Patients in the HIGH C3a cohort exhibited significantly higher systemic inflammation (CRP; p = 0.041), higher complement activation (C3a and C5a: p < 0.001), and more pronounced endothelial glycocalyx damage, as reflected by reduced eGC height (− 44%, p < 0.01) and elevated Syndecan-1 levels (+ 203%, p < 0.01).

These alterations were associated with impaired endothelial function, reduced nitric oxide bioavailability (− 34%, p = 0.033), prolonged hospitalization (p < 0.01), and lower left ventricular ejection fraction, indicating a more severe clinical phenotype in patients with high C3a levels.

These results suggest that, although the groups had comparable demographic and traditional cardiovascular risk profiles, the HIGH C3a group was characterized by a greater degree of systemic and vascular inflammation, endothelial injury, and complement activation. This was associated with prolonged hospitalization and reduced ventricular function. In contrast, patients in the LOW C3a group demonstrated more preserved endothelial structure and lower inflammatory activity. The STEMI group exhibited an intermediate profile in most parameters.

### Differences between LOW and HIGH C3a groups

**Fig. 1:**
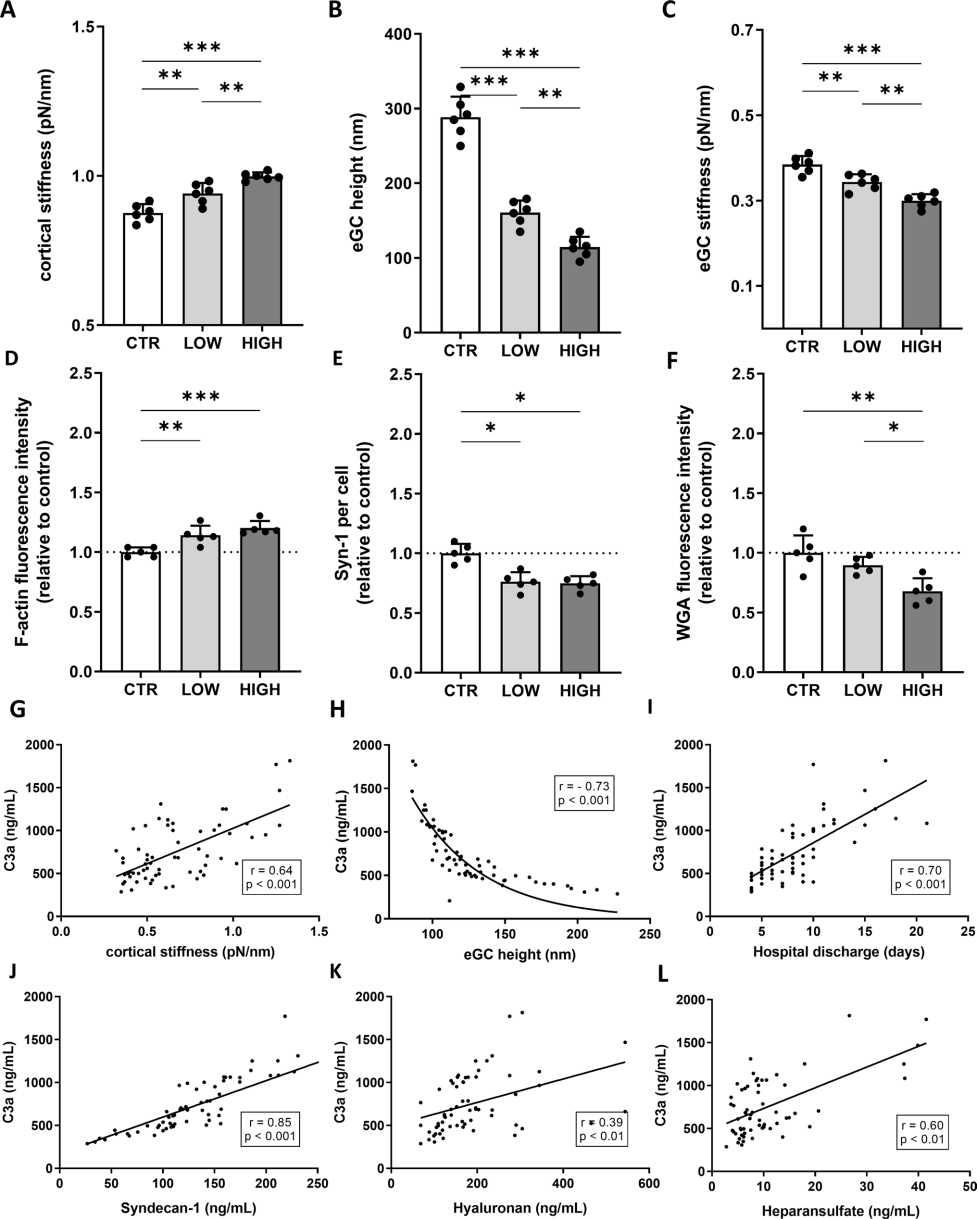
Structural and Mechanical Alterations of the Endothelial Glycocalyx Across Disease Severity. Statistical analysis of atomic force microscopy (AFM) nanoindentation measurements of human umbilical vein endothelial cells (HUVEC) monolayers. Data showing mean ± SD of **A** cortical stiffness, **B** endothelial glycocalyx (eGC) height and **C** eGC stiffness. Each dot represents a single cell measurement (N = 6). **D**–**F** Statistical fluorescence intensity analysis relative to the control group of **D** F-actin stained HUVEC monolayers relative to control group. **E** Syndecan-1 antibody-stained HUVEC monolayers. Graphs showing the amount of syndecan-1 per cell relative to control group. **F** Wheat germ agglutinin (WGA)-stained HUVEC monolayers. Graphs showing the measured fluorescence intensity relative to control group (D-F: N = 5). **G–L** Correlation of C3a concentrations in STEMI patient sera vs. cortical stiffness, endothelial glycocalyx (eGC) height, days until discharge from hospital, syndecan-1, heparan sulfate, and hyaluronan (Rho (r) and p-values (p) shown for correlations). Groups: LOW: STEMI sera of the lowest C3a concentration quartile; HIGH: STEMI sera of the highest C3a concentration quartile; CTR: healthy donors. p-values: ***p < 0.001; **p < 0.01; *p < 0.05

Direct comparison of LOW and HIGH C3a cohorts revealed marked differences in endothelial integrity, complement activation, and vascular inflammation (Table 1; Fig. 1). The most pronounced differences were observed in endothelial glycocalyx structure and nanomechanical properties.

**Table 1:** Baseline demographic and disease characteristics

Characteristic		STEMI	LOW	HIGH	p-values
Baseline characteristics					
Age (years)		64 (12)	62 (13)	61 (11)	0.736
Male sex (%)		76.2	87.5	75.0	0.135
Hypertension (%)		79.0	75.0	86.7	0.411
Diabetes (%)		20.6	12.5	18.8	0.626
Obesity (%)		27.0	25.0	31.3	0.694
BMI (kg/m^2^)		27.4 (4.5)	27.3 (3.9)	26.8 (6.4)	0.808
Pos. family history (%)		86.7	89.0	75.0	0.197
Hyperlipidaemia (%)		88.9	93.8	81.3	0.285
Smoking (%)		62.3	62.5	71.4	0.605
Renal insufficiency (%)		39.7	48.8	43.8	0.354
Clinical characteristics					
Heart rate (beats/min)		102 (15)	100 (19)	106 (16)	0.358
RR systolic (mmHg)		165 (14)	181 (14)	179 (18)	0.706
RR diastolic (mmHg)		74 (14)	79 (15)	81 (16)	0.820
LMS stenosis (%)		11.1	6.3	12.5	0.544
LAD stenosis (%)		74.6	81.3	68.8	0.414
LCX stenosis (%)		46.0	56.3	58.2	0.465
RCA stenosis (%)		34.9	62.5	68.8	0.710
LVEF (grouped; %)	< 40%	15.6	16.7	30.0	0.676
	40–50%	37.8	50.0	45.0	0.674
	> 50%	44.4	33.3	25.0	0.389
Days until discharge from hospital		8.4 (3.7)	6 (2)	13 (4)	**< 0.001**
Laboratory values					
Troponin max. (pg/mL)	[Ref.: 0–14]	3053.0 (2653)	1391 (2309)	1696 (2600)	0.728
CK max. (U/L)	[Ref.: 20–200]	1100.8 (1464)	847 (1093)	728.75 (794)	0.727
LDH max. (U/L)	[Ref.: 135–225]	446.6 (318.9)	315.93 (183)	499.07 (360)	0.094
Pro BNP II (pg/mL)	[Ref.: 0–121]	1972.7 (2614)	1001 (1581)	3788 (4530)	0.206
Creatinine (mg/dL)	[Ref.: 0.7–1.2]	1.18 (0.9)	1.17 (0.43)	1.01 (0.27)	0.221
GFR (mL/min/1,7)	[Ref.: 80–140]	72.1 (23.3)	69.25 (25.8)	75.31 (20.2)	0.465
HbA1c (%)	[Ref.: 0–6]	6.0 (1.0)	6.02 (0.99)	6.08 (0.95)	0.867
CRP max. (mg/L)	[Ref.: 0–5]	45.4 (48.0)	38.14 (32.2)	65.35 (70.9)	**0.041**
Leukocytes (Gpt/L)	[Ref.: 4–9]	17.41 (4.6)	12.39 (4.1)	13.29 (6.9)	0.657
Erythrocytes (Tpt/L)	[Ref.: 4.5–5.5]	4.6 (0.6)	4.66 (0.6)	4.76 (0.6)	0.599
Thrombocytes (Gpt/L)	[Ref.: 150–400]	252.1 (72.1)	270.50 (91.9)	258.31 (64.7)	0.668
Na^+^ (mmol/L)	[Ref.: 136–145]	135.7 (3.7)	136.06 (2.3)	134.63 (4.9)	0.302
K^+^ (mmol/L)	[Ref.: 3.5–5.1]	4.3 (0.7)	4.16 (0.5)	4.40 (0.5)	0.218
Ca^2+^ (mmol/L)	[Ref.: 2.15–2.55]	2.25 (0.11)	2.29 (0.09)	2.17 (0.15)	0.110
HDL (mg/dL)	[Ref.: 55–150]	55.5 (11.7)	46.68 (12.3)	38.47 (12.5)	0.088
LDL (mg/dL)	[Ref.: 0–150]	128.2 (37.8)	120.13 (30.7)	102.38 (37.6)	0.183
Triglycerides (mg/dL)	[Ref.: 0–200]	168.9 (64.2)	111.50 (66.6)	101.38 (48.3)	0.640
**Endothelial and glycocalyx values**					
eGC height (nm)		125.9 (32.8)	170.60 (30.5)	96.25 (5.9)	**< 0.001**
eGC stiffness (pN/nm)		0.34 (0.05)	0.41 (0.02)	0.27 (0.01)	**< 0.001**
Syndecan-1 (ng/mL)		136.72 (69.3)	72.15 (27.96)	218.85 (81.02)	**< 0.001**
Heparan sulfate (ng/mL)		10.82 (8.6)	6.65 (2.4)	18.75 (13.1)	**< 0.01**
Hyaluronic acid (µg/mL)		182.9 (85.9)	168.41 (61.3)	175.24 (69.3)	0.770
Nitric oxide (mM)		6.39 (2.3)	8.6 (2.6)	5.7 (1.9)	**0.033**
Angiopoetin-2 (ng/mL)		18.5 (8.7)	17.27 (7.26)	14.52 (4.30)	0.204
C3a (ng/mL)		676.0 (343.4)	409.71 (65.71)	1223.0 (254.00)	**< 0.001**
C5a (ng/mL)		36.2 (27.0)	26.19 (10.53)	81.50 (28.14)	**< 0.001**

*BMI* body mass index, *BNP* brain natriuretic peptide, *C3a* Complement factor C3a, *C5a* Complement factor C5a, *CK* creatine kinase, *CRP* c-reactive protein, *eGC* endothelial glycocalyx, *GFR* glomerular filtration rate, *HbA1c* haemoglobin A1c (glycated haemoglobin) , *HDL* high density lipoprotein, *HIGH* highest quartile of C3a, *LAD* left anterior descending artery, *LCX* left circumflex artery, *LDH* lactate dehydrogenase, *LDL* low density lipoprotein, *LMS* left main stem, *LOW* lowest quartile of C3a, *LV EF* left ventricular ejection fraction, *Pos. family history* positive family history of myocardial infarction, *RCA* right coronary artery, *Ref*. laboratory reference values, *RR* (Riva-Rocci) blood pressure, *STEMI* ST-elevation myocardial infarction

p-values (LOW vs. HIGH): Pearson-Chi-square test (categorical data), group differences were analysed using Student’s t-test (for parametric values) or Mann–Whitney-U test (for nonparametric values). Data in mean ± standard deviation (m ± SD); categorical data in percentage (%)

Patients in the HIGH C3a group showed a significantly reduced eGC height (− 44%, p < 0.001) and stiffness (− 34%, p < 0.01) compared with the LOW group (Fig. 1A–C). Circulating markers of glycocalyx shedding were substantially elevated, with Syndecan-1 increased more than threefold (+ 203%, p < 0.001) and heparan sulfate nearly tripled (+ 182%, p < 0.01) (Table 1).

Complement activation was markedly enhanced in the HIGH cohort, as reflected by increased C3a (+ 198%, p < 0.001) and C5a concentrations (+ 211%, p < 0.01). These changes were accompanied by significantly reduced nitric oxide bioavailability (− 34%, p = 0.033) and longer hospitalization (+ 117%, p < 0.001)(Table 1).

Together, these data indicate that elevated C3a levels are associated with pronounced endothelial glycocalyx degradation, impaired endothelial function, and adverse clinical parameters.

### Structural and mechanical alterations of the endothelial glycocalyx across disease severity

Confluent HUVEC monolayers were stimulated with patient or control sera and the nanomechanical properties (height and stiffness) of the endothelial surface layers (eGC and cortex) were probed by using the AFM nanoindentation technique. Comparisons were made between healthy controls and the previously described LOW and HIGH cohorts (Fig. 1).

Cortical stiffness increased stepwise from CTR to LOW (+ 7%, p < 0.01) and HIGH (+ 14%, p < 0.001), indicating enhanced cytoskeletal tension with increasing disease severity (Fig. 1A). In parallel, eGC height was markedly reduced in the LOW (− 44%) and HIGH (− 60%) cohorts compared with controls (both p < 0.001) (Fig. 1B). eGC stiffness decreased by 11% in LOW and 22% in HIGH patients (p < 0.001), indicating substantial softening of the glycocalyx with increasing disease burden (Fig. 1C).

Consistently, these nanomechanical changes were accompanied by increased cortical F-actin polymerization (+ 14% in LOW, + 18% in HIGH; p < 0.001) and a significant reduction in glycocalyx-associated fluorescence signals, including Syndecan-1 (− 24% and − 25%; p < 0.05) and WGA staining (− 10% and − 29%; p < 0.01) (Fig. 1D–F).

Correlation analyses revealed strong associations between circulating C3a levels and eGC height (r = − 0.736, p < 0.001), Syndecan-1 shedding (r = 0.856, p < 0.001), cortical stiffness (r = 0.645, p < 0.001), and hospitalization duration (r = 0.709, p < 0.001) (Fig. 1G–L). Collectively, these data identify a pattern of associations between complement activation, glycocalyx degradation, cortical stiffening, and actin reinforcement in the endothelium of severely affected patients.

### Complement C3a directly induces cortical remodeling and glycocalyx degradation

To examine whether C3a directly mediates endothelial biomechanical and structural changes, cells were exposed to increasing concentrations of recombinant human C3a (0, 50, 250, 500, and 1000 ng/mL) and different stimulation durations (0, 30, 60, and 120 min and 24 h). The nanomechanical properties (height and stiffness) of the cellular cortex and the eGC of HUVEC monolayers were quantified via AFM-based nanoindentation.

**Fig. 2:**
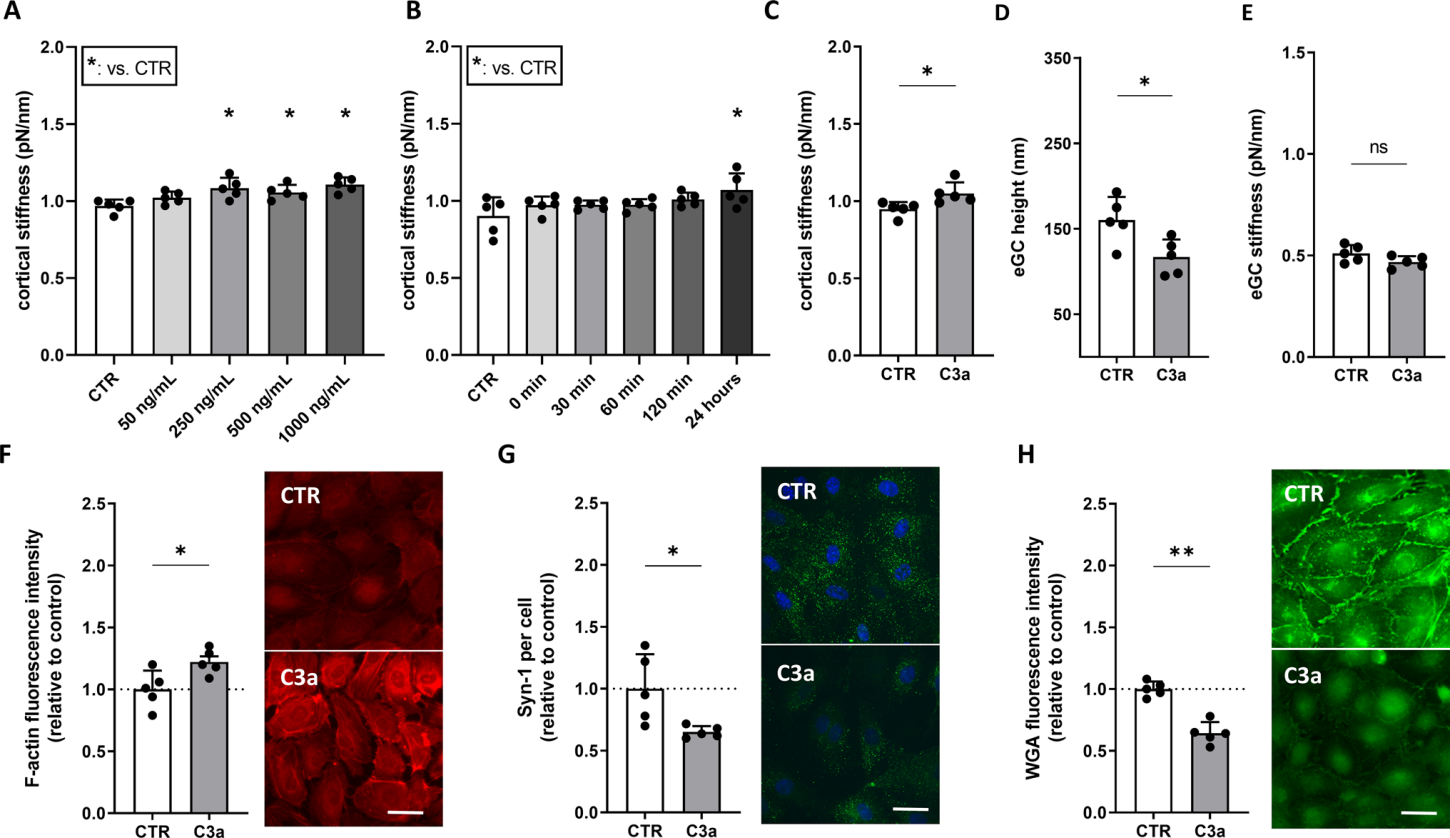
Complement C3a Directly Induces Cortical Remodeling and Glycocalyx Degradation. Different concentrations of C3a as well as different stimulation durations were tested for their influence on cortical stiffness measured via atomic force microscopy (AFM) nanoindentation technique. Statistical analysis of AFM measurements of human umbilical vein endothelial cells (HUVEC) monolayers. Data showing mean ± SD of **A** cortical stiffness after stimulation with different concentrations of C3a (0, 50, 250, 500 and 1000 ng/mL; N = 5). **B** Cortical stiffness after stimulation with 250 ng/mL of C3a using different stimulation durations (0, 30, 60, and 120 min and 24 h; N = 5). Statistical analysis of **C** cortical stiffness, **D** eGC height, and **E** eGC stiffness after stimulating HUVEC monolayers with 250 ng/mL of C3a for 24 h (N = 5). **F–H** Representative fluorescence images and statistical fluorescence intensity analyses. **F** F-actin stained HUVEC monolayers after stimulation as described (red: F-actin) relative to the control. **G** Syndecan-1 Antibody-stained HUVEC monolayers (green: syndecan-1; blue: cell nucleus) after stimulation. Graphs showing the amount of syndecan-1 per cell relative to the control. **H** Wheat germ agglutinin (WGA)-stained HUVEC monolayers after stimulation as described (green: WGA; representing unspecific eGC components) showing fluorescence intensity relative to the control group. (**F**–**H**: N = 5; scale bars: 50 μm). Groups: CTR: stimulation with standard cell culture media; C3a: cell culture media + 250 ng/mL of C3a. p-values: ***p < 0.001; **p < 0.01; *p < 0.05.; ns: not significant

C3a induced a dose-dependent increase in cortical stiffness, with significant effects observed at concentrations ≥ 250–1000 ng/mL (all; p < 0.05) and maximal stiffening at 250 ng/mL (+ 10%, p < 0.05) (Fig. 2A). Time-course analyses revealed significant cortical stiffening after 24 h (p < 0.05), but not at earlier time points (Fig. 2B). Based on these results, a stimulation concentration of 250 ng/mL and a stimulation time of 24 h were selected for the following experiments.

At 250 ng/mL C3a, detailed AFM and fluorescence analyses showed profound endothelial alterations.

Cortical stiffness increased by 7% (p < 0.05), while eGC height was reduced by 24% (p < 0.05) (Fig. 2C, D). This was accompanied by increased F-actin polymerization (+ 9%, p < 0.05) and marked loss of glycocalyx components, with Syndecan-1 (p < 0.05) and WGA fluorescence reduced to 65% and 67% of control levels, respectively (p < 0.01) (Fig. 2F–H).

### C3a receptor antagonism prevents C3a-induced cortical stiffening and glycocalyx degradation

Treatment with patient serum or recombinant C3a reduced eGC and stiffened the cellular cortex. C3aRA (SB290157) was used to study the C3a:C3aR axis influences on vascular surface and eGC integrity. Two experimental approaches were employed to examine the impact of C3aRA: (i) Stimulation with CTR versus C3a (250 ng/mL), both with and without C3aRA treatment. (ii) Stimulation with serum from STEMI patients versus serum from healthy controls, both with and without C3aRA treatment.

**Fig. 3:**
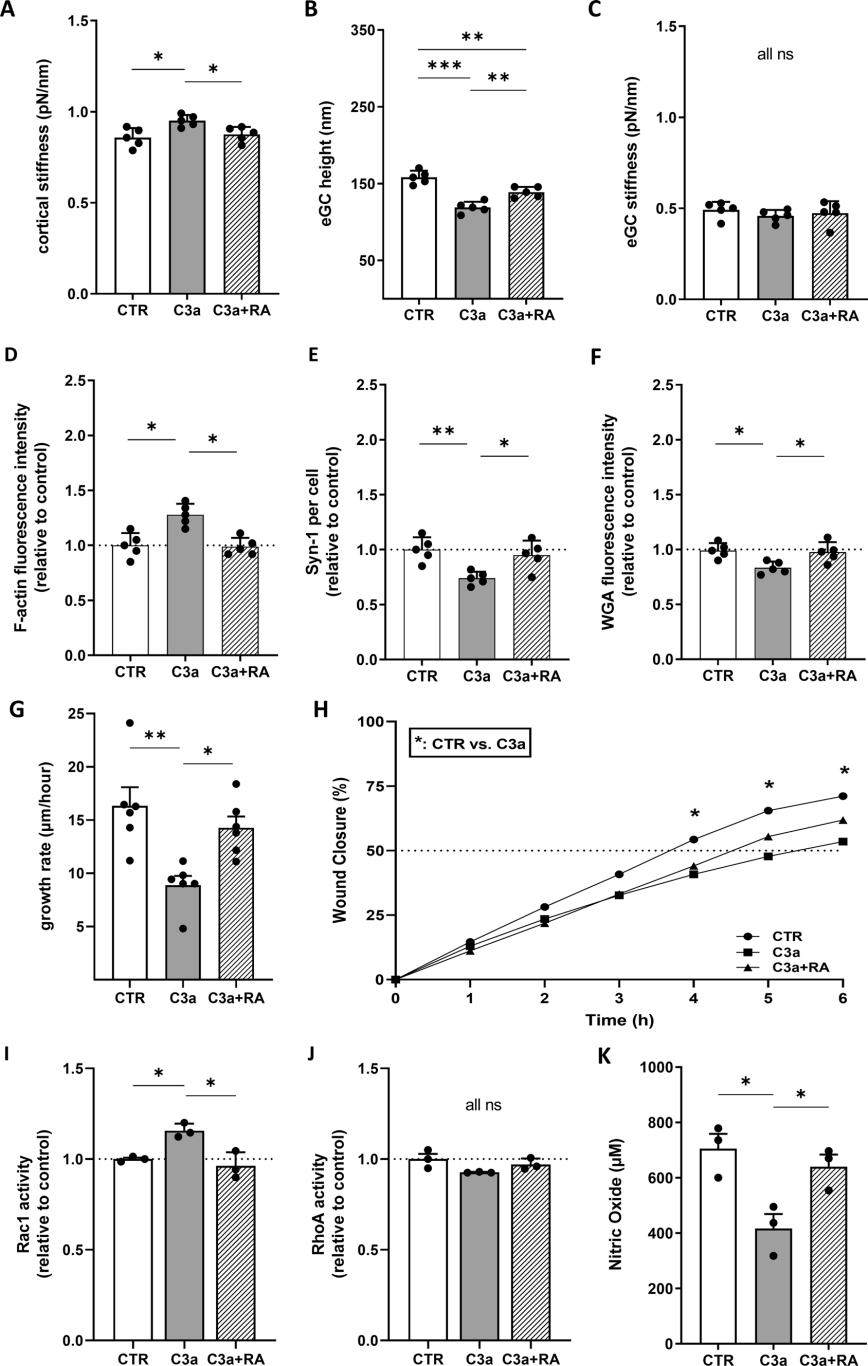
C3a receptor antagonism (RA) prevents C3a-induced cortical stiffening and glycocalyx degradation. Statistical analysis of atomic force microscopy (AFM) nanoindentation measurements of human umbilical vein endothelial cells (HUVEC) monolayers Data showing mean ± SD of **A** cortical stiffness, **B** endothelial glycocalyx (eGC) height, and **C** eGC stiffness of CTR, C3a, and C3a + RA groups.; N = 5) as described in detail in the methods section. **D**–**F** Statistical fluorescence intensity analyses. **D** F-actin stained HUVEC monolayers relative to control group. **E** Syndecan-1 antibody-stained HUVEC monolayers after stimulation. Graphs showing the amount of syndecan-1 per cell relative to control group. **F** Wheat germ agglutinin (WGA)-stained HUVEC monolayers. Graphs showing the measured fluorescence intensity relative to control group (**D**–**F**: N = 5). **G**–**H** Statistical evaluation of wound healing assays (N = 6). Data showing **G** the growth rate (μm/h) as described in the methods section as well as **H** wound closure in % (*: CTR vs. C3a). **I** Small GTPases activation analysis of Rac1 from confluent HUVEC. Bar graphs show raw optical density (O.D.) measured with an absorbance wavelength of 490 nm relative to control (N = 3). **J** Small GTPases activation analysis of RhoA from confluent HUVEC after treatment. Bar graphs show raw optical density (O.D.) measured with an absorbance wavelength of 490 nm relative to control (N = 3). **K** Statistical analysis of nitric oxide (NO) concentrations. NO concentration was measured chemiluminescence-based via NOA-280i (N = 3). Groups: CTR: stimulation with standard cell culture media; C3a: cell culture media + 250 ng/mL of C3a; C3a + RA: cell culture media + 250 ng/mL of C3a + C3a-Receptor antagonist (SB290157; 1:1000). p-values: ***p < 0.001; **p < 0.01; *p < 0.05.; ns: not significant

Pharmacological inhibition of C3aR using SB290157 effectively prevented C3a-induced cortical stiffening (+ 10.8% under C3a vs. n.s. with C3aRA; p < 0.05) and partially restored eGC height (− 12% vs. CTR; p < 0.01) (Fig. 3A, B). C3a-induced actin polymerization (+ 27.9%) and glycocalyx loss were fully reversed by C3aR antagonism (Fig. 3D–F).

To investigate the migration characteristics of HUVEC monolayers after C3a treatment or stimulation with STEMI serum, wound healing assays were performed. Functionally, C3a impaired endothelial wound closure in scratch assays by 53% (p < 0.01), while RA co-treatment restored migration capacity (Fig. 3G). Wound closure (%/h) was compared among all groups. Both CTR and C3a+ RA groups reached 50% wound closure after approximately 4.5 h. Wound closure was significantly further advanced after 4 h in controls compared to those in the C3a stimulation group (p < 0.05) (Fig. 3H).

Collectively, these findings demonstrate that C3a receptor blockade effectively counteracts C3a-mediated cortical stiffening, glycocalyx disruption, and impaired endothelial migration, underscoring the key role of C3a–C3aR signaling in cytoskeletal-glycocalyx coupling.

To determine the pathway of cortical stiffening, small GTPases were quantified. For this, Rac1 and RhoA activities were measured as key mechanotransduction players, regulating actin dynamics, using G-LISA assays. C3a stimulation led to a significant activation of Rac1 (p < 0.05), consistent with actin remodeling. RA co-treatment completely abolished these effects, returning Rac1 GTPase activity to baseline (p < 0.05 vs. C3a; n.s. vs. CTR) (Fig. 3I). RhoA showed no enhanced activity after stimulation with both, C3a or STEMI serum. There were no significant effects after RA treatment for both groups (Fig. 3J).

To evaluate endothelial function to the different stimuli, NO concentrations were measured, as indicator of endothelial function and homeostasis. Functionally, NO bioavailability was reduced by C3a by 38% and restored by C3aR blockade (Fig. 3K). These findings indicate that C3aR antagonism suppresses cytoskeletal activation through Rac1 inhibition and restores endothelial NO bioavailability, reversing the C3a-mediated alterations.

### C3a receptor blockade reverses endothelial dysfunction induced by STEMI patient serum

**Fig. 4:**
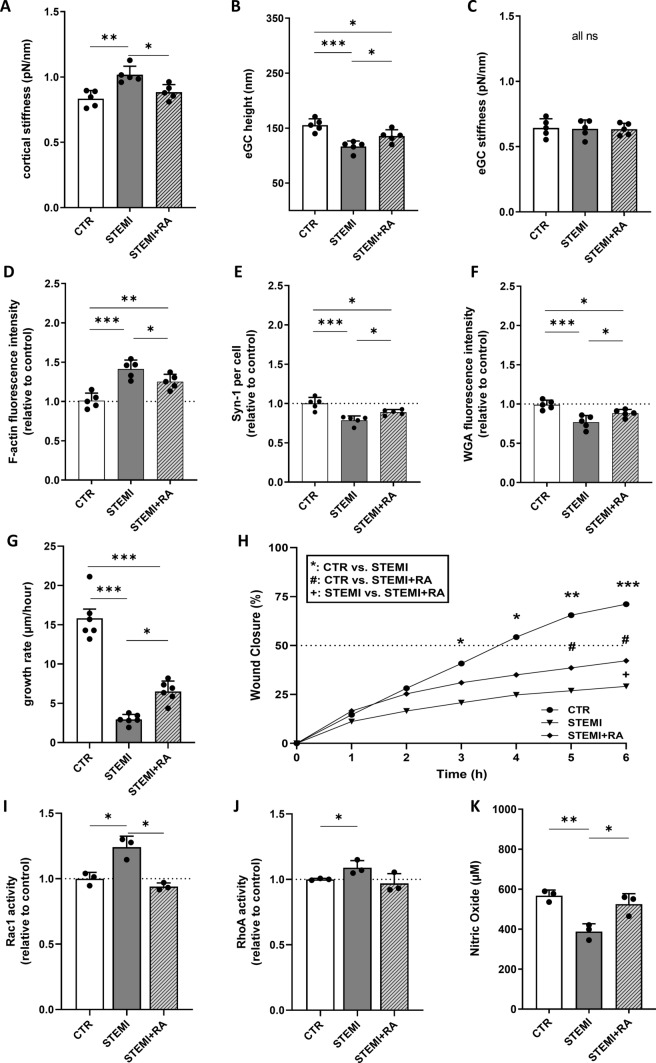
C3a receptor blockade reverses endothelial dysfunction induced by STEMI patient serum. Statistical analysis of atomic force microscopy (AFM) nanoindentation measurements of human umbilical vein endothelial cells (HUVEC) monolayers. **A**–**C** Data showing mean ± SD of **A** cortical stiffness, **B** endothelial glycocalyx (eGC) height, and **C** eGC stiffness of CTR, STEMI, and STEMI + RA groups. Each dot represents a single cell measurement (8 force-distance curves per dot; N = 5) as described in detail in the methods section. **D**–**F** Statistical fluorescence intensity analyses. **D** F-actin stained HUVEC monolayers relative to control group. **E** Syndecan-1 antibody-stained HUVEC monolayers after stimulation. Graphs showing the amount of syndecan-1 per cell relative to control group. **F** Wheat germ agglutinin (WGA)-stained HUVEC monolayers. Graphs showing the measured fluorescence intensity relative to control group (D-F: N = 5). **G**–**H** Statistical evaluation of wound healing assays for CTR, STEMI, and STEMI + RA groups (N = 6). Data showing **G** the growth rate (μm/h) as described in the methods section as well as **H** wound closure in % (*: CTR vs. C3a; #: CTR vs. STEMI + RA; + : STEMI vs. STEMI + RA). **I** Small GTPases activation analysis of Rac1 from confluent HUVEC. Bar graphs show raw optical density (O.D.) measured with an absorbance wavelength of 490 nm relative to control (N = 3). **J** Small GTPases activation analysis of RHoA from confluent HUVEC after treatment. Bar graphs show raw optical density (O.D.) measured with an absorbance wavelength of 490 nm relative to control (N = 3). **K** Statistical analysis of nitric oxide (NO) concentrations. NO concentration was measured chemiluminescence-based via NOA-280i (N = 3). Groups: CTR: serum of healthy donors; STEMI: serum of STEMI patients; STEMI + RA: serum of STEMI patients + C3a-Receptor antagonist (SB290157; 1:1000). p-values: ***p < 0.001; **p < 0.01; *p < 0.05.; ns: not significant

Exposure of endothelial cells to serum from STEMI patients reproduced key structural, mechanical, and functional alterations observed after stimulation with recombinant C3a (Fig. 4). Compared with control serum, STEMI serum induced a significant increase in cortical stiffness (+ 17.8%, p < 0.01) and a pronounced reduction in endothelial glycocalyx height (− 22.6%, p < 0.001), indicating endothelial stiffening and surface layer degradation (Fig. 4A, B). eGC stiffness remained unchanged (Fig. 4C).

These nanomechanical changes were accompanied by marked cytoskeletal remodeling, as reflected by a strong increase in cortical F-actin fluorescence (+ 39.8%, p < 0.001), and by significant loss of glycocalyx-associated markers, including Syndecan-1 (− 19%, p < 0.001) and WGA staining (− 13%, p < 0.001) (Fig. 4D–F).

Endothelial migration was severely impaired by STEMI serum (CTR vs. STEMI: − 83%; p < 0.001). RA significantly improved migration rates (p < 0.001 vs. CTR; p < 0.05 vs. STEMI) but did not reach control levels (Fig. 4G). After 3.5 h wound closure reached 50% in the control group. Neither STEMI nor STEMI + RA groups reached the 50% closure mark during the experiment. There was a significant difference between the STEMI and the RA group after 6 h (p < 0.05) indicating that wound closure was partially restored by RA (Fig. 4H). These data show that C3a receptor inhibition partially protects against STEMI serum-induced endothelial stiffening, actin reorganization, and eGC loss, suggesting that complement activation contributes substantially to endothelial dysfunction in STEMI.

In the STEMI model, Rac1 activity was significantly increased compared with controls (CTR vs. STEMI, p < 0.05) and was restored to baseline by receptor antagonism (STEMI vs. RA, p < 0.05; CTR vs. STEMI + RA, n.s.) (Fig. 4I). In contrast, RhoA activity showed a nonsignificant trend toward elevation following STEMI stimulation (Fig. 4J). Exposure to STEMI serum reduced endothelial NO release (− 29%; p < 0.01), whereas C3aR blockade rescued NO levels (p < 0.05 vs. STEMI) (Fig. 4K). Together, these results indicate that C3a-dependent signaling contributes substantially to the endothelial structural and functional dysfunction induced by STEMI patient serum, although additional serum-derived inflammatory mediators are likely involved.

### C3a receptor inhibition reduces monocyte adhesion and binding forces at the endothelial interface

**Fig. 5:**
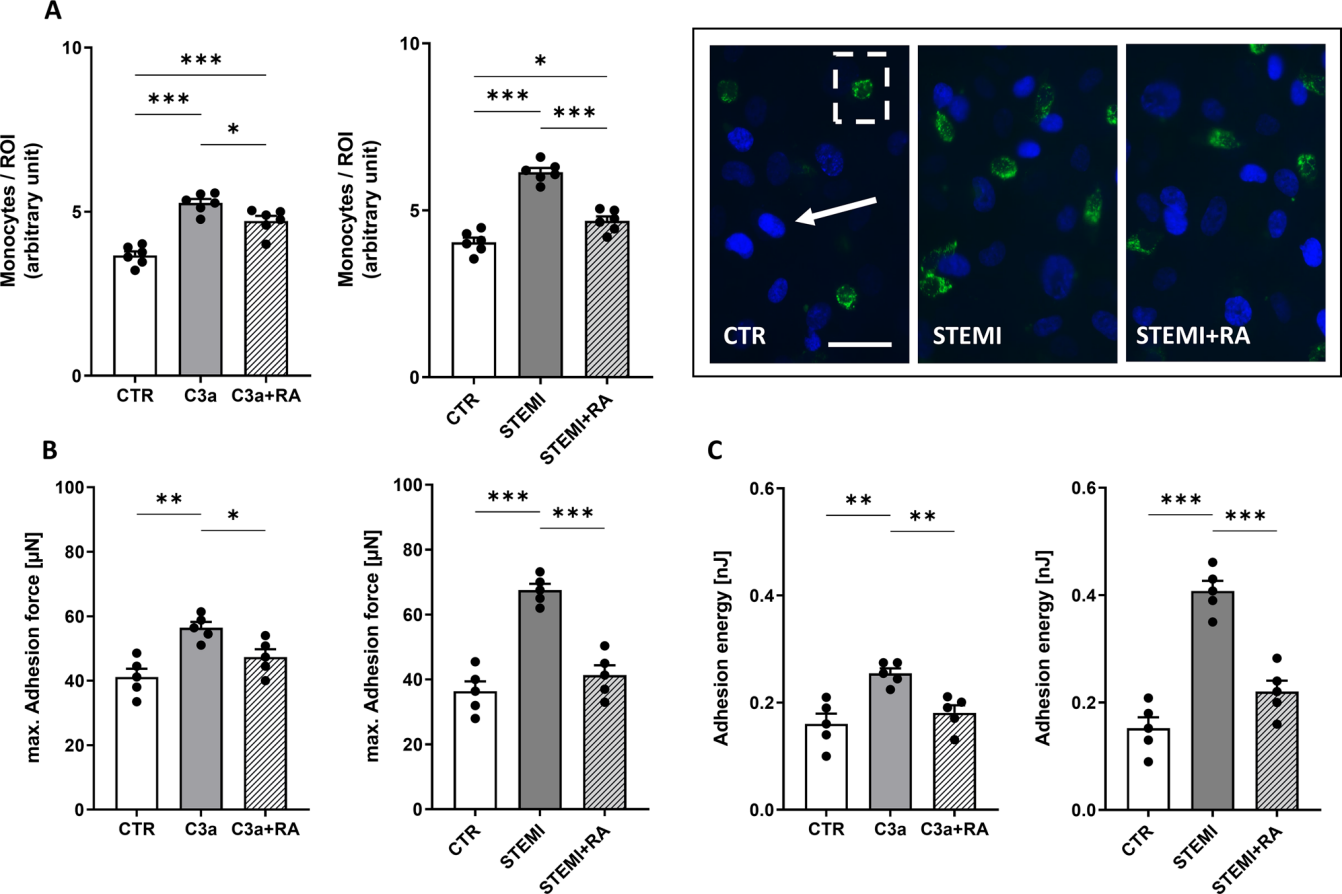
C3a receptor inhibition reduces monocyte adhesion and binding forces at the endothelial interface. Data showing mean ± SD of monocyte adhesion to human umbilical vein endothelial cells (HUVEC) monolayers measured via monocyte-wash-away assay and atomic force microscopy (AFM)-based single-cell force spectroscopy (SCFS). **A** Statistical analysis of adherent monocytes per region of interest (ROI) quantified via monocyte-wash-away assay with previous CD14-staining (N = 6). Representative fluorescence images of human monocytes (green: CD14-labeled monocytes; indicated by white dashed box) adhesive to HUVEC monolayer (blue: HUVEC nuclei; indicated by white arrow; scale bar: 30 μm). **B** max. Adhesion force (in µN) between monocyte and HUVEC monolayer (max. adhesion force between monocyte and HUVEC monolayer) (N = 5) measured via AFM-based SCFS. **C** Adhesion energy (in nJ) between monocyte and HUVEC monolayer (energy needed to separate monocyte from HUVEC) (N = 5) measured via AFM-based SCFS. Groups: CTR: stimulation with standard cell culture media; C3a: cell culture media + 250 ng/mL of C3a; C3a + RA: cell culture media + 250 ng/mL of C3a + C3a-Receptor antagonist (SB290157; 1:1000). CTR: serum of healthy donors; STEMI: serum of STEMI patients; STEMI + RA: serum of STEMI patients + C3a-Receptor antagonist (SB290157; 1:1000). p-values: ***p < 0.001; **p < 0.01; *p < 0.05

In the wash-away adhesion assay, C3a significantly increased monocyte attachment (+ 43.6%; p < 0.001), which was attenuated by RA (p < 0.05)(Fig. 5A). Likewise, STEMI serum enhanced monocyte adhesion (+ 54%; p < 0.001), whereas RA markedly reduced binding (p < 0.001 vs. STEMI) (Fig. 5A). These findings indicate that C3a receptor blockade effectively suppresses the pro-adhesive endothelial phenotype induced by C3a or STEMI serum, limiting both adhesion strength and monocyte recruitment.

Single-cell force spectroscopy revealed a pronounced increase in monocyte adhesion following C3a and STEMI stimulation. Maximum adhesion forces were elevated by both C3a (+ 37%; p < 0.01) and STEMI serum (+ 87%; p < 0.001) compared with controls, whereas RA restored adhesion forces to baseline (n.s. vs. CTR) (Fig. 5B). Similarly, total adhesion energy increased after C3a stimulation by + 59% (p < 0.01), which was prevented by RA. STEMI serum likewise elevated adhesion energy (p < 0.001), an effect significantly mitigated by RA (p < 0.001 vs. STEMI) (Fig. 5C).

All pro-adhesive effects were significantly attenuated by C3aR antagonism, restoring adhesion parameters to near-control levels. Collectively, these results demonstrate that monocyte adhesion is tightly linked to endothelial activation, with enhanced binding reflecting a pro-inflammatory, dysfunctional endothelial state that can be effectively reversed by C3a receptor antagonism.

### Rac-1-inhibition reverses endothelial dysfunction induced by C3a and STEMI serum in HUVEC and HCMEC

**Fig. 6:**
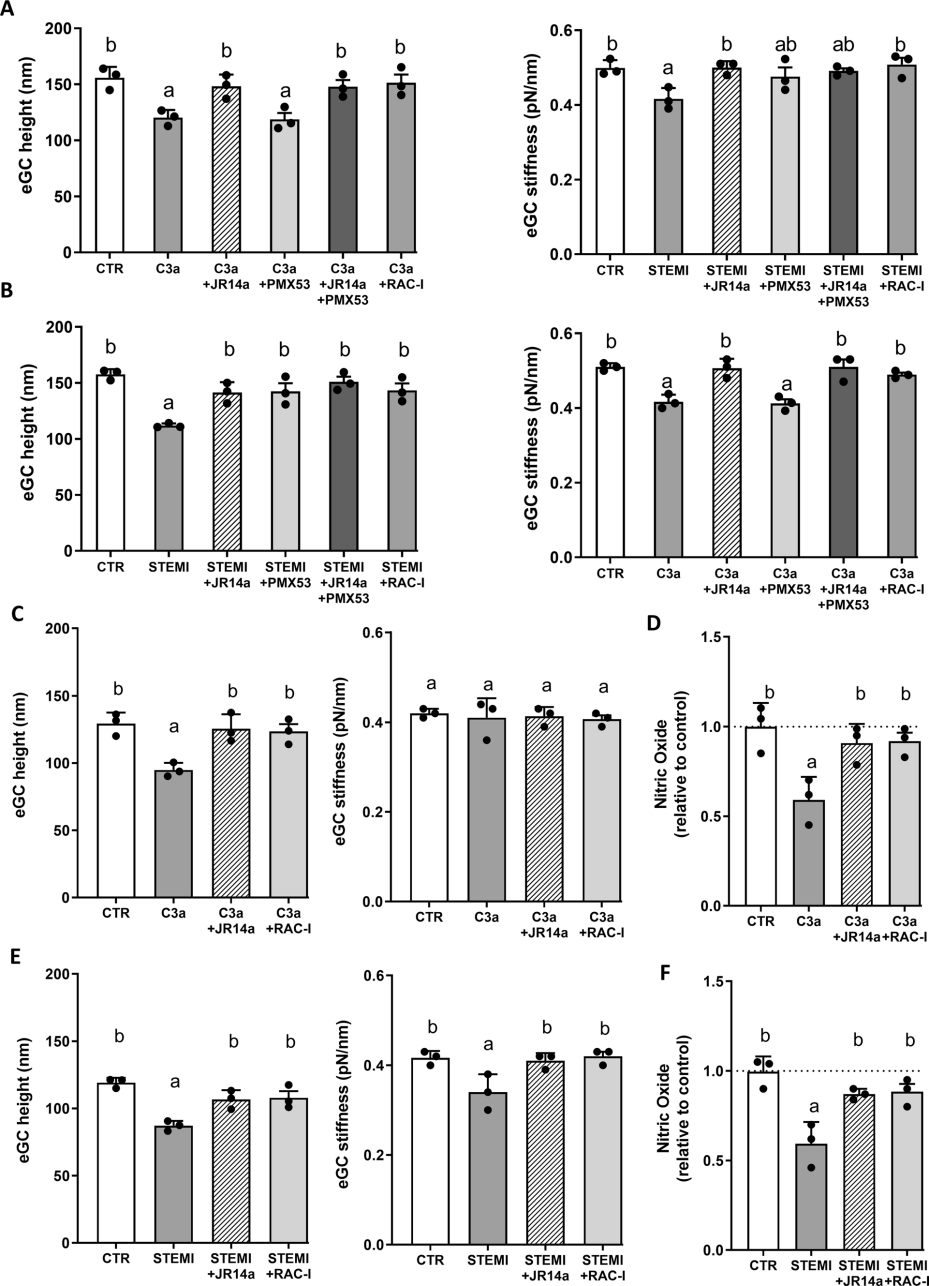
Rac1 inhibition reverses endothelial dysfunction induced by C3a and STEMI serum in HUVEC and HCMEC. **A** Endothelial glycocalyx (eGC) height and stiffness in HUVEC after stimulation with recombinant C3a and pharmacological inhibition of C3aR1 (JR14a), C5aR1 (PMX53), combined C3aR1 + C5aR1 inhibition, and Rac1 inhibition. C3a significantly reduced glycocalyx height compared with control, which was prevented by C3aR1 inhibition and Rac1 inhibition but not by C5aR1 inhibition. Reduced eGC stiffness indicates shedding. **B** eGC height and stiffness in HUVEC after incubation with serum from STEMI patients. STEMI serum significantly reduced glycocalyx height compared with healthy control serum. C3aR1 inhibition and Rac1 inhibition prevented glycocalyx loss, while C5aR1 inhibition partially restored glycocalyx height. Combined C3aR1 and C5aR1 inhibition further improved glycocalyx preservation. **C** Glycocalyx height in human cardiac microvascular endothelial cells (HCMEC) after stimulation with recombinant C3a and pharmacological inhibition of C3aR1 and Rac1. C3a-induced glycocalyx degradation was prevented by both C3aR1 inhibition and Rac1 inhibition. **D** Nitric oxide (NO) bioavailability in HCMEC after stimulation with recombinant C3a with or without C3aR1 inhibition and Rac1 inhibition. **E** Glycocalyx height in HCMEC after incubation with STEMI serum. STEMI serum reduced glycocalyx height compared with control serum, which was attenuated by C3aR1 inhibition and Rac1 inhibition. **F** Nitric oxide (NO) bioavailability in HCMEC after incubation with STEMI serum with or without C3aR1 inhibition and Rac1 inhibition. Reduced NO levels induced by C3a and STEMI serum were restored by both C3aR1 inhibition and Rac1 inhibition. Data are presented as mean ± SD (all N = 3). Statistical analysis was performed using one-way ANOVA with Tukey post hoc test. Different letters indicate significant differences between groups (p < 0.05)

To further dissect the signaling pathways involved in complement-mediated endothelial dysfunction, Rac1 inhibition, C3a receptor antagonism, and C5a receptor antagonism were evaluated, including combined C3aR and C5aR inhibition. Endothelial glycocalyx height and nitric oxide bioavailability were measured in HUVEC and HCMEC endothelial cells (Fig. 6).

Stimulation of HUVECs with recombinant C3a resulted in a significant reduction in endothelial glycocalyx (eGC) height compared with control cells (p = 0.01) corresponding to an approximate 30% decrease compared with control conditions (Fig. 6a). Inhibition of C3aR1 using JR14a prevented the C3a-induced glycocalyx loss, with glycocalyx height not significantly different from control. Similarly, Rac-1 inhibition prevented glycocalyx degradation. In contrast, inhibition of C5aR1 using PMX53 did not prevent glycocalyx loss, and glycocalyx height remained significantly reduced compared with control (p = 0.007). Combined inhibition of C3aR1 and C5aR1 did not result in additional protective effects compared with C3aR1 inhibition alone. Furthermore, C3a-treated cells differed significantly from JR14a-treated cells (p = 0.047), from combined JR14a + PMX53 treatment (p = 0.048), and from Rac-1 inhibition (p = 0.025), further supporting the involvement of the C3aR1-Rac1 pathway (Fig. 6a).

Exposure of HUVECs to STEMI patient serum significantly reduced glycocalyx height compared with serum from healthy controls (p < 0.001), corresponding to an approximate reduction in glycocalyx height of 40% (Fig. 6b). Treatment with the C3aR1 antagonist JR14a prevented glycocalyx loss (p = 0.25 vs. control), and similar protective effects were observed with Rac-1 inhibition (p = 0.37 vs. control). In STEMI, inhibition of C5aR1 significantly restored glycocalyx height (p = 0.0094 vs. STEMI). Combined inhibition of C3aR1 and C5aR1 further improved glycocalyx preservation and reached control levels (p = 0.0013 vs. STEMI; p = 0.92 vs. CTR). STEMI serum differed significantly from all inhibitor-treated groups (JR14a: p = 0.012; PMX53: p = 0.009; Rac-1 inhibitor: p = 0.007) (Fig. 6b).

#### Effects of C3a on glycocalyx height and stiffness in HCMECs

In human cardiac microvascular endothelial cells (HCMEC), stimulation with recombinant C3a significantly reduced glycocalyx height compared with control cells (p = 0.005), corresponding to an approximate reduction of 30% (Fig. 6c). This effect was prevented by C3aR1 inhibition (p = 0.94 vs. control) and by Rac-1 inhibition (p = 0.84 vs. control). C3a differed significantly from both JR14a-treated cells (p = 0.01) and Rac-1 inhibited cells (p = 0.015) (Fig. 6c).

Stimulation with STEMI patient serum significantly reduced glycocalyx height in HCMECs compared with control serum (p = 0.018), corresponding to an approximate reduction of 25% (Fig. 6e). This reduction was prevented by C3aR1 inhibition (p = 0.13 vs. control) and Rac-1 inhibition (p = 0.18 vs. control). Both, stimulation with recombinant C3a and STEMI serum led to a reduced NO bioavailability in HCMEC supernatants. This effect was prevented by both, C3aR1 inhibition as well as by Rac-1 inhibition (both p < 0.05) (Fig. 6d, f).

Taken together, these results demonstrate that both recombinant C3a and factors present in STEMI patient serum induce endothelial glycocalyx loss and increased endothelial stiffness via a C3aR1-dependent Rac1 signaling pathway in both macrovascular and microvascular endothelial cells.

## Discussion

The present study demonstrates that activation of the complement cascade, specifically via the C3a:C3aR-axis, contributes significantly to endothelial dysfunction and glycocalyx degradation in the setting of STEMI. By integrating human patient data with mechanistic in vitro experiments, we demonstrate a correlation between elevated C3a levels and significant alterations of the eGC, increased cortical actin polymerization, reduced NO bioavailability, and enhanced monocyte adhesion. All of these are decisive markers for endothelial dysfunction and inflammatory processes in the cardiovascular system. In the context of contemporary concepts of myocardial infarction, our findings align with the emerging paradigm that coronary microvascular injury is a major determinant of infarct severity and clinical outcome. Recent reviews emphasize that endothelial dysfunction, microvascular inflammation, and impaired vasodilatory capacity persist despite timely reperfusion and substantially contribute to infarct expansion and post-infarction heart failure. By demonstrating a strong association between C3a activation, glycocalyx degradation, and endothelial dysfunction, our data provide a mechanistic framework linking early complement activation to microvascular pathology in STEMI [32, 36].

Thus, the present findings support an association between early complement activation and endothelial injury in patients with acute myocardial infarction, while providing mechanistic evidence from in vitro experiments, thereby supporting the hypothesis that C3a-mediated signaling directly impairs endothelial surface nanomechanics and barrier function.

### Complement activation and endothelial injury in STEMI

Complement activation is considered to be one of the earliest and most potent inflammatory responses following ischemia–reperfusion injury in myocardial infarction [5, 22]. In the event of myocardial necrosis, damage-associated molecular patterns and altered cell surface structures activate the classical, lectin, and alternative pathways, converging at the cleavage of C3 into the anaphylatoxin C3a and the opsonin C3b [49, 53]. The resulting local increase in C3a initiates proinflammatory signaling within the vascular endothelium, leading to enhanced expression of adhesion molecules, leukocyte recruitment, and disruption of vascular homeostasis [18, 40]. In accordance with prior studies on the C5a/C5aR1 axis[46], our findings expand this concept upstream, showing that elevated C3a levels are associated with endothelial injury in patients, while cell-based experiments demonstrate that C3a stimulation is sufficient to induce structural and functional endothelial damage.

Compared to controls and patients of the LOW-Cohort the patients in the HIGH-C3a cohort exhibited significantly higher glycocalyx degradation, as indicated by reduced eGC height and stiffness and elevated circulating levels of syndecan-1 and heparan sulfate. These markers indicate significant shedding of proteoglycan and glycosaminoglycan components, signifying endothelial surface erosion [7, 50]. The inverse correlation between serum C3a concentrations and eGC height further supports the hypothesis that there is a direct relationship between complement activation and endothelial structural deterioration. Notably, these patients exhibited diminished NO bioavailability and prolonged hospitalization, indicating an association between biochemical injury markers and clinical outcomes. While our study focuses on C3a signaling, it is important to recognize that other complement effectors, such as C5a, are also activated in parallel during acute myocardial infarction and may contribute to vascular and endothelial injury. Recent data show that increased C5a concentrations in STEMI patients associate with significant endothelial glycocalyx shedding, cortical stiffening, reduced NO bioavailability, and enhanced leukocyte adhesion, effects that are attenuated by C5aR1 antagonism. Inclusion of C5a-related mechanisms provides a more comprehensive view of complement-driven vascular injury and highlights that C3a may represent one of several interacting complement effectors rather than an exclusive driver of endothelial dysfunction[46]. Thus, our findings are consistent with prior research demonstrating that endothelial glycocalyx degradation impairs mechanotransduction, reduces NO production, and promotes microvascular dysfunction[38, 44]. An important limitation of the present study is the limited specificity of C3a as an independent effector in the clinical STEMI cohort. Although elevated C3a levels were strongly associated with endothelial dysfunction and glycocalyx degradation, other complement components—particularly C5a—were activated in parallel, indicating a broader complement response. Due to the observational nature of the human data and the absence of multivariable modeling or selective complement inhibition in patients, it cannot be conclusively determined whether C3a represents a causal driver or a surrogate marker of global complement activation in vivo. Therefore, causality is only supported by mechanistic in vitro experiments, whereas the clinical findings should be interpreted as associative and hypothesis-generating.

A potential limitation of other pharmacological studies targeting the C3a receptor relates to the specificity of SB290157, which has been reported to exhibit partial agonistic and off-target effects in certain experimental settings. To address the specificity of C3aR1-mediated signaling and to exclude potential off-target effects of SB290157, additional pharmacological experiments were performed using a second C3aR1 antagonist (JR14a), a Rac1 inhibitor (NSC23766), and a C5aR1 antagonist (PMX53). These experiments confirmed a central role of the C3a-C3aR1-Rac1 signaling axis in endothelial glycocalyx degradation and endothelial stiffening.

Both recombinant C3a stimulation and incubation with STEMI patient serum resulted in significant glycocalyx height reduction, which was reversed not only by SB290157 but also by JR14a. Importantly, inhibition of Rac1 similarly prevented glycocalyx loss and endothelial stiffening, indicating that Rac1 acts downstream of C3aR1 signaling in mediating cytoskeletal remodeling and glycocalyx damage. The loss-of-function experiments using Rac1 inhibition demonstrate that Rac1 activity is not merely associated with C3a-induced endothelial changes but is functionally required for cytoskeletal remodeling, glycocalyx degradation, and impaired nitric oxide bioavailability, thereby placing Rac1 as a central downstream mediator of C3aR signaling in endothelial dysfunction.

However, stimulation with STEMI serum likely reflects a more complex complement activation pattern than isolated C3a stimulation. During STEMI, both C3a and C5a levels are elevated. C5a acts via C5a receptor 1 and activates RhoA-dependent signaling pathways, promoting the transition from G-actin to F-actin, resulting in cortical stiffening and subsequent glycocalyx degradation[46]. Thus, both complement-derived anaphylatoxins may contribute to endothelial mechanical and structural alterations during myocardial infarction.

In our experiments, inhibition of C5aR1 using PMX53 alone did not fully prevent glycocalyx degradation or endothelial stiffening, whereas C3aR1 inhibition showed stronger protective effects. However, combined inhibition of C3aR1 and C5aR1 showed the most consistent protective effects, suggesting that both pathways contribute to endothelial injury in the context of STEMI, with C3aR1 signaling appearing to be the dominant pathway under the conditions studied.

These findings may also explain why therapeutic strategies targeting only the C5 pathway have shown limited protective effects in myocardial infarction, as upstream C3a-mediated endothelial injury may persist despite C5 downstream inhibition[23, 33, 43, 46].

Taken together, these additional pharmacological experiments strengthen the mechanistic interpretation of our findings and suggest that endothelial glycocalyx degradation and cortical stiffening in response to STEMI serum are mediated by a combination of C3aR1-Rac1 and C5aR1-RhoA signaling pathways, with a predominant contribution of C3aR1-dependent signaling. The functional consequences of glycocalyx degradation extend beyond endothelial dysfunction and directly impact coronary microvascular perfusion. Loss of glycocalyx integrity promotes capillary leakage, leukocyte adhesion, and microvascular plugging, thereby aggravating no-reflow phenomena and sustained ischemia. Recent comprehensive analyses underscore that persistent microvascular dysfunction is a key contributor to adverse ventricular remodeling and progression toward ischemic heart failure. In this regard, our observation that elevated C3a levels are associated with both glycocalyx loss and prolonged hospitalization highlights the clinical relevance of complement-mediated endothelial surface injury in STEMI [2, 28].

### From C3a/C3aR activation to cytoskeletal remodeling

Our results suggest that C3a-induced Rac1 activation is a key driver of cortical actin remodeling, and evidence of a possible further mechanistic sequence can be conclusively verified by previous studies. Binding of C3a to its G-protein-coupled receptor (C3aR) signals predominantly via Gi/o-type G-proteins and β-arrestin-dependent pathways[4, 39] leading to Gβγ-mediated phosphoinositide-3-kinase γ (PI3Kγ) activation and conversion of phosphatidylinositol-4,5-bisphosphate (PIP2) to phosphatidylinositol-3,4,5-trisphosphate (PIP3) [9, 42]. PIP3 recruits Rac1-specific guanine nucleotide exchange factors (GEFs), including P-Rex1 and Tiam1, promoting GDP-GTP exchange and localized Rac1 activation at the endothelial cortex [19, 26, 51].

Activated Rac1 regulates actin dynamics through the WAVE complex, promoting Arp2/3-mediated F-actin formation[11, 16], and via p21-activated kinases (PAK)-mediated LIM kinases (LIMK) phosphorylation, which inactivates Cofilin, a major actin-depolymerizing protein [3, 41].

These mechanisms collectively drive the polymerisation of actin. Accordingly, activation of the C3a receptor can lead to cortical stiffening and stress fibre formation, which are consistent with our AFM and fluorescence data. Notably, all changes were reversed by C3aR blockade, suggesting that the C3a–C3aR–Rac1 axis may contribute to endothelial cytoskeletal remodeling and glycocalyx degradation.

### Rac1-Dependent cytoskeletal changes and glycocalyx damage

The observed endothelial dysfunction can be explained by the connection between cortical actin reorganization and glycocalyx degradation. The eGC and the submembranous actin cortex form a functional mechanical unit in which transmembrane proteoglycans (e.g., Syndecan-1, Syndecan-4) and hyaluronan receptors (CD44) serve as physical linkers between the glycocalyx and the actin cytoskeleton [1, 13]. Increased Rac1 activity has been shown to enhance actin polymerization, thereby increasing cortical stiffness and promoting shedding of glycocalyx components [14, 48]. These mechanical effects are frequently accompanied by the activation of matrix metalloproteinases (MMPs) and heparanases, which enzymatically cleave heparan sulfate and hyaluronan chains, exacerbating glycocalyx loss [6, 34]. As a result, C3a-mediated activation of Rac1 initiates the physical detachment of the glycocalyx, which leads to its functional impairment.

This loss of glycocalyx integrity was found to compromise the mechanotransductive capacity of the endothelium, leading to reduced shear stress sensing and impaired activation of endothelial NO synthase (eNOS) [31, 54]. Consequently, NO bioavailability declines, as confirmed in our experiments, where both recombinant C3a and STEMI patient serum decreased NO levels, while C3aR inhibition restored them. Given the pivotal role of NO in vascular tone regulation and anti-inflammatory signaling, its reduction represents a key step to endothelial dysfunction [50]. Furthermore, we could demonstrate, that wound healing was restored after C3a antagonism. Endothelial wound healing is essential after myocardial infarction, as impaired endothelial repair contributes to microvascular dysfunction and adverse cardiac remodeling. This process is critical for restoring vascular integrity and limiting ischemia-induced injury [17].

### Endothelial activation and monocyte adhesion

Beyond structural changes, C3a-induced Rac1 activation drives a proadhesive endothelial phenotype, as evidenced by our monocyte adhesion assays. Endothelial glycocalyx breakdown increases the accessibility of adhesion molecules, supporting leukocyte–endothelial adhesion and interaction [15]. The enhanced monocyte binding forces observed under C3a or STEMI serum stimulation were reversed by C3aR antagonism, indicating that this effect depends on intact C3aR signaling. These findings are consistent with prior reports showing that complement activation is associated with enhanced leukocyte recruitment and transmigration in vivo [12, 55], while our in vitro data demonstrate a direct, C3a-dependent effect on endothelial–monocyte interactions.

The sequence of events, which includes C3a activation, Rac1 signaling, actin polymerization, glycocalyx shedding, reduced NO bioavailability, and monocyte adhesion, describes a mechanistic cascade linking early complement activity to endothelial dysfunction after myocardial infarction. This model may explain why downstream complement blockade (e.g., C5 or C5a inhibition) has shown incomplete protection in clinical trials or basic research [23, 33, 46], since C3a-driven mechanisms occur upstream and independently of terminal pathway activation. From a therapeutic perspective, recent reviews have questioned the effectiveness of targeting terminal complement components alone in myocardial infarction, given that upstream complement activation initiates endothelial and microvascular injury early after ischemia. Inhibition at the level of C3 or C3a signaling has been proposed as a strategy to simultaneously attenuate inflammation, preserve endothelial barrier function, and protect the coronary microcirculation. Our findings support this concept by demonstrating that C3a receptor antagonism restores endothelial nanomechanics, nitric oxide bioavailability, and leukocyte adhesion, thereby addressing key mechanisms of microvascular dysfunction highlighted in recent translational research [2, 36].

## Conclusion

The present data indicate that elevated C3a levels are associated with endothelial dysfunction and glycocalyx degradation in STEMI patients, while mechanistic in vitro experiments demonstrate that activation of the C3a:C3aR-Rac1 axis directly mediates endothelial cytoskeletal remodeling and glycocalyx damage, driving cortical stiffening and endothelial dysfunction, which can be improved by C3aR blockade. These findings highlight a potential therapeutic strategy to preserve vascular integrity and mitigate complement-driven endothelial injury during STEMI, providing a mechanistic basis for future C3a-targeted interventions.

## Data Availability

The dataset examined in this study is available upon reasonable request from the corresponding author.
